# A New Proposal for Learning Curve of TEP Inguinal Hernia Repair: Ability to Complete Operation Endoscopically as a First Phase of Learning Curve

**DOI:** 10.1155/2014/528517

**Published:** 2014-04-23

**Authors:** Mustafa Hasbahceci, Fatih Basak, Aylin Acar, Orhan Alimoglu

**Affiliations:** ^1^Department of General Surgery, Faculty of Medicine, Bezmialem Vakif University, Vatan Street, Fatih, 34093 Istanbul, Turkey; ^2^Department of General Surgery, Umraniye Education and Research Hospital, Umraniye, 34766 Istanbul, Turkey; ^3^Department of General Surgery, Lutfi Kirdar Kartal Education and Research Hospital, Kartal, 34890 Istanbul, Turkey; ^4^Department of General Surgery, Faculty of Medicine, Istanbul Medeniyet University, Kadikoy, 34722 Istanbul, Turkey

## Abstract

*Background*. The exact nature of learning curve of totally extraperitoneal inguinal hernia and the number required to master this technique remain controversial. * Patients and Methods*. We present a retrospective review of a single surgeon experience on patients who underwent totally extraperitoneal inguinal hernia repair. * Results*. There were 42 hernias (22 left- and 20 right-sided) in 39 patients with a mean age of 48.8 ± 15.1 years. Indirect, direct, and combined hernias were present in 18, 12, and 12 cases, respectively. The mean operative time was 55.1 ± 22.8 minutes. Peritoneal injury occurred in 9 cases (21.4%). Conversion to open surgery was necessitated in 7 cases (16.7%). After grouping of all patients into two groups as cases between 1–21 and 22–42, it was seen that the majority of peritoneal injuries (7 out of 9, 77.8%, *P* = 0.130) and all conversions (*P* = 0.001) occurred in the first 21 cases. * Conclusions*. Learning curve of totally extraperitoneal inguinal hernia repair can be divided into two consequent steps: immediate and late. At least 20 operations are required for gaining anatomical knowledge and surgical pitfalls based on the ability to perform this operation without conversion during immediate phase.

## 1. Introduction

Totally extraperitoneal (TEP) inguinal hernia repair has gained popularity in the recent two decades since the first introduction in 1992 by Dulucq [1]. It offers a hernia repair of minimal incisions with more favorable postoperative course including less pain and quicker return to work especially more pronounced in bilateral inguinal hernia [2]. However, this technique requires specialized anatomical knowledge, two-hand manipulation for reduction of hernia sac, and mesh placement within a limited working space. Therefore, acceptance and implementation of this technique have been slow compared to the adoption of other minimal invasive procedures such as cholecystectomy [3, 4].

In addition to the technical dexterity, there are some drawbacks for the common adoption of this technique including increased operative times, complications during the early learning curve, and almost absolute necessity for general anesthesia [5, 6]. Consequently, the learning curve of TEP inguinal hernia repair for the inexperienced surgeons carries paramount importance. However, the exact nature of learning curve and the number required to master the technique are still focus of a debate.

There are a limited number of studies evaluating the learning curve for TEP inguinal hernia repair [2, 3, 7, 8]. Although there were some numerical suggestions beginning from 20 cases, the required number of operation to fulfill the learning curve has been reported even 250 repairs to fully master all aspects of the TEP approach [2, 3, 6, 9]. However, instead of recognizing the learning curve as a solid piece, it could be separated into two phases in order to ease the implementation and evaluation:* immediate* as an initial phase of ability to complete the operation and* late* as a latter phase of performing TEP with good outcomes.

In the present study, we try to evaluate the minimum required number of cases from the beginning of the learning curve to complete the operation as TEP inguinal hernia repair without conversion in the absence of supervision from an experienced endoscopic hernia surgeon.

## 2. Patients and Methods

A retrospective demographic, clinical, and operative data collection of adult patients who underwent TEP inguinal hernia repair between December 2011 and May 2012 was performed from a prospectively held database. Written consent was taken from each patient for both TEP and Lichtenstein inguinal hernia repairs for the cases in which conversion might be required. The patients with American Society of Anesthesiologists (ASA) classes IV and V, who had contraindications for general anesthesia, previous open, or laparoscopic lower abdominal surgery except open inguinal hernia repair, with emergency admission for complicated inguinal hernia, with femoral hernia diagnosed by imaging techniques, and who were unwilling to be operated by TEP inguinal hernia repair, were excluded.

All TEP repairs were performed under general anesthesia by a single surgeon (MH) who had a satisfactory experience with laparoscopic cholecystectomy and who performed more than 500 Lichtenstein inguinal hernia repair previously. For TEP inguinal hernia repair, active participation to the operations (*n* > 10) performed by an experienced surgeon was done.

Patients' demographics, body mass index (kg/m^2^), ASA class, features of the hernias, operative findings including time, presence of peritoneal injury, conversion to open surgery, and cause for the conversion, complications within the postoperative 30 days, and length of hospital stay were documented prospectively into a computerized database.

Operation time was calculated as the time from the first incision to the last suture. Complications were grouped as intraoperative including bleeding from epigastric or testicular arteries, peritoneal, testicular, or nerve injuries, and postoperative including hematoma or seroma formation, urinary retention treated by catheterization, neuralgia, wound infection, and early recurrence during the first 30 days. Hematoma or seroma was defined as an accumulation of blood or fluid in the subcutaneous tissues from the umbilicus to the scrotum. Neuralgia was defined as a pain in the inguinal region and medial aspect of the thigh occurred after the operation. Wound infection was defined as occurrence of redness with or without drainage from the incisions. In the absence of hematoma and seroma, any swelling in the inguinal region verified by clinical examination and imaging techniques was defined as early recurrence. Length of stay was calculated as the number of days in the hospital after the surgery. Patients were seen within the fourth week postoperatively.

### 2.1. Operative Technique

Patients were asked to empty their urinary bladder just before the operation. No prophylactic antibiotics were administered. Under general anesthesia, anterior rectus sheath on the side of inguinal hernia was incised via infraumbilical incision. Then, a space was created below the rectus without incising the posterior rectus sheath. In case of bilateral inguinal hernia, the entrance was done on the dominant side. After formation of a tunnel with the help of blunt-tipped instruments, 10 mm trocar was introduced and carbon dioxide insufflation was started with a maximum pressure of 15 mmHg. Balloon dissectors were not used.

The optical telescope with 0 degree was inserted and blunt dissection by gentle side-to-side movements was performed until the symphysis pubis was clearly seen. The inferior epigastric vessels were clearly visualized laterally on the posterior surface of the rectus muscle. The retropubic space of Retzius and the space of Bogros were easily expanded by this telescopic approach. Two 5 mm trocars were introduced between the umbilicus and the symphysis pubis. The hernia defect was identified. Dissection of the peritoneal sac from the cord structures in cases of laterally placed indirect inguinal hernia or retraction from the abdominal wall defect in cases of medially placed direct inguinal hernia or both in cases of combined inguinal hernia were performed. Dissection of indirect inguinal hernia sac was completed either by reduction or transection in which it was closed by metallic clips. Peritoneal defects were closed either by metallic clips or suturing. After appropriate dissection of all potential hernia spaces medially from the symphysis pubis laterally to the psoas muscle and reduction of the hernia sac(s), a polypropylene mesh (Prolene, Ethicon, LCC) with a diameter of 15 × 10 cm was inserted and placed over the entire musculopectineal orifice with sufficient overlap at the medial and lateral borders. No keyhole over the mesh or no fixation of the mesh was being used. After the complete desufflation under permanent visual control of the operative area, removal of the trocars was performed. One fascial suture to subumbilical incision was applied. Skin incisions were closed in an appropriate manner. In case of difficulty or in the event of a complication, the operation was converted to Lichtenstein inguinal hernia repair in all cases.

### 2.2. Statistical Analysis

Statistical calculations were performed using NCSS (Number Cruncher Statistical System, 2007) and PASS (Power Analysis and Sample Size) Statistical software (Utah, USA, 2008). Normally distributed continuous variables were expressed as mean ± standard deviation (SD). The range including minimum and maximum values was also added. Categorical variables were expressed as frequencies and percentages of an appropriate denominator. The Student's *t*-test was used for analysis of normally distributed, descriptive continuous variables, which were expressed as mean ± SD. The Chi-Square Test and Fischer's Exact Test were used to compare qualitative variables. Differences were considered statistically significant, if the *P* value was equal to or less than 0.05.

## 3. Results

The study group included 38 male and one female patient with 42 hernias. The mean age and body mass index of the patients were 48.8 ± 15.1 years (range from 19 to 73 years) and 26.2 ± 3.4 kg/m^2^ (range from 19 to 32 kg/m^2^), respectively. ASA classes I, II, and III distribution of the patients was 25, 15, and 2, respectively.

There were 22 left- and 20 right-sided hernias. Indirect, direct, and combined hernias were present in 18, 12, and 12 cases, respectively. Hernias with previous repairs were detected only in 4 cases. Peritoneal injury occurred in 9 cases (21.4%). Conversion to open surgery was necessitated in 7 cases (16.7%). There was no bleeding and testicular or nerve injury intraoperatively. The mean operative times were 55.1 ± 22.8 minutes (range from 20 to 110 minutes) excluding the patients with conversion to open surgery. The causes for conversion were summarized in Table 1.

Occurrence of peritoneal injury was not related with the age and BMI of the patient, type and side of hernia, and presence of previous repair (*P* > 0.05 for all). Conversion occurred significantly in right-sided (*P* = 0.041) and recurrent hernias (*P* = 0.011). No significant differences were detected between age and BMI of the patients and type of the hernia and conversion (*P* > 0.05 for all).

All patients were grouped into two groups: Groups I and II consisted of the cases between 1–21 and 22–42, respectively (Table 2). Two groups were similar with regard to age, BMI, and operation time. Although peritoneal injury occurred more frequently in Group I (33.3% versus 9.5%), it did not reach statistical significance (*P* = 0.130). However, all conversions were seen in Group I (*P* = 0.009).

All patients were discharged at the first day postoperative. Postoperative urinary retention, neuralgia, and wound infection were not seen. However, in three patients, two in Group I and one in Group II, seroma formation was detected and managed conservatively. There was one early recurrence in Group I. No mortality was seen.

## 4. Discussion

The learning curve has been defined as the minimum number of operations required for gaining adequate knowledge of pitfalls and technical factors leading stabilization of operation times and complication rates [3, 9]. In literature, there were several cut-off values for the learning period of endoscopic hernia repair up to 250 cases which was regarded as comfort zone [6, 10]. In a Cochrane review, it was suggested to perform at least between 30 and 100 operations as a critical threshold level to become an experienced surgeon [10, 11]. It is generally accepted that for a recurrence rate of less than 1%, more than 60 cases under supervision were recommended [2, 10, 12]. Lau et al. reported that at least 80 operations were required for the mean operation time of less than 1 hour [3]. It was also shown that even after more than 400 individually performed TEP procedures, there was a progress in reducing the conversion rate, the incidence of short-term complications, and the operative times [10]. These findings suggested the necessity of a rather long learning curve for TEP procedures.

In previous studies, operation time less than 1 hour has been regarded as one of the parameters used to state the learning curve precisely [3, 4]. However, it is possible to perform this operation in a time period of less than one hour even in the beginning period, as in the present study. Gaining experience to use the minimal invasive techniques in other aspects of surgery might help to implement the technique in short time with greater efficiency. However, mastering the technique mandates not only finishes the operation in short time without conversion but also performs the operation with low recurrence rates. It could be helpful to separate two phases of learning curve as* immediate* and* late.* Therefore, we and others propose that an inexperienced beginner surgeon should perform at least 20 cases in accordance with the principles of endoscopic TEP inguinal hernia repair to become a familiar surgeon [9]. The exact number for becoming an experienced surgeon which is most probably more than 20 cases should be evaluated with future prospective studies.

Perceived pressure of the surgeons to complete the operations expediently was thought to be responsible for the high conversion rate which has been frequently experienced during endoscopic TEP inguinal hernia repair with an incidence of 2%–17% [8, 13]. Although our conversion rate during the first 21 cases was higher, we did not encounter any conversion during the second part of this study in accordance with Lal's findings [7]. Some authors have mentioned that more than 50 cases were required for the surgeons who were unfamiliar with preperitoneal space [7]. However, adequate perception of the preperitoneal anatomy with careful dissection can be gathered during the first 20 cases without causing any morbidity according to the present study.

Appropriate patient selection has been shown to be an important parameter for the success of the operation during early period. Irreducible hernias, hernias in patients with previous lower quadrant surgery, have been excluded in several early TEP series [3, 14]. Certain patient characteristics including female gender, higher BMI, previous history of abdominal surgery, and scrotal and bilateral hernias were also shown to be important for the high risk of conversion and intraoperative complications even for experienced surgeons. However, liberal inclusion of the patients in to the study including recurrent and sliding hernias was applied during the learning curve of this study which might affect our high conversion rate. It could be possible to diminish the conversion rate in our study, if the strict inclusion criteria were used. Indeed, it is recommended to select relatively younger and slender male patients less than 60 years of age with unilateral, nonscrotal primary inguinal hernia during the learning period for TEP inguinal hernia repair [8, 14].

It has been also shown that the presence of an experienced endoscopic hernia surgeon or performance of previous Stoppa's procedures prevents unnecessary recurrences caused by surgical errors and helps overcome the difficulty which has been experienced during the learning period [7, 8]. Experience with preperitoneal space anatomy is the most important factor for performing the posterior approaches either through open or endoscopic approaches [7, 15]. Therefore, performance of previous Stoppa's procedure might also be unhelpful for a surgeon who is unfamiliar to this space. Therefore, it is believed that active participation to endoscopic TEP inguinal hernia repairs performed by an experienced surgeon can facilitate the transition to TEP procedures [4, 9, 12].

Peritoneal injury has been regarded as the most important operative complication to cause the loss of exposure in a limited preperitoneal area [8]. It has been reported that the occurrence of this complication can be seen in almost half of the cases [16]. In the present study, peritoneal injury occurred in 21.4% of the cases and was regarded as the reason for conversion in two out of seven conversions. Thus, use of nontraumatic graspers and scissors with cautery is advised to avoid such complication during dissection of the operative area and reduction of the indirect hernia sac.

Preperitoneal dissection can be performed by disposable balloon dissectors or by the help of 0° telescopes [17]. The balloon dissector has been known to decrease the operation time and to reduce conversion rates [13, 18]. Therefore, it is recommended to use such instruments especially during the early period in the learning curve besides its high cost. However, these instruments were not favored in the present study because of the financial considerations though their beneficial effect. Blunt dissection by using 0° telescopes can be easy, if the entrance to the preperitoneal space can be succeeded through cleavage of the posterior lamina of transversalis fascia. We recommend dissecting the preperitoneal space by using telescopes only in accordance with the precautions published before [15].

During endoscopic TEP inguinal hernia repair, it is important to dissect all possible hernia sites to prevent the recurrences. The short-term recurrences were most likely due to technical errors causing improper identification of the indirect hernia sac [3, 8]. Although there was only one short-term recurrence in our series, inadequate dissection causing missed indirect hernia was thought to be responsible for early recurrence. Therefore, it is advised to isolate the cord structures at least for a distance of 4 cm to dissect the all defective areas and to deflate the air under direct vision to overcome such technical problems. For prevention of the direct recurrences, extensive lateral preperitoneal dissection and good positioning of the mesh with sufficient size covering the Hasselbach triangle is recommended [3, 7, 8].

This study has some limitations including its retrospective design with small number of cases and lack of the long-term follow-up. The main objective of this study was to measure the minimum number of endoscopic TEP inguinal hernia repairs to complete the operation without any conversion for a beginner surgeon. Therefore, we did not include several operative outcomes including long-term recurrence and postoperative pain into the aims of this study, although these parameters are the most important endpoints for a successful evaluation of an endoscopic hernia repair [8].

Our results were derived from a single teaching hospital and from a single surgeon experience. Although there may be some difficulty to generalize our findings because of the individual differences based on skill set and training structure, they can be regarded as a baseline level for the minimum requirement for TEP inguinal hernia repair.

## 5. Conclusion

The learning curve of TEP inguinal hernia repair can be divided in two consequent steps: the* immediate* which shows the technical experience to accomplish endoscopic surgery without complications and conversions and the* late* to become an experienced surgeon with a late recurrence rate of less than 1%. At least 20 operations are required for gaining anatomical knowledge of preperitoneal space and surgical pitfalls based on the ability to perform the operation without conversion.

## Figures and Tables

**Table 1:** Causes for conversion.

Reason	Number
Peritoneal injury causing loss of exposure	2
Difficulty to determine the anatomy	2
Adhesions caused by previous hernia repair	2
Sliding hernia	1

**Table 2:** Demographic and operative data of the groups.

Parameter	Groups		*P*
	Group I (numbers 1–21)	Group II (numbers 22–42)	
Age^*β*^ (years)	47.0 ± 18.0 (19–73)	50.6 ± 11.6 (27–69)	0.434
BMI^*β*^(kg/m^2^)	25.9 ± 3.8 (19–32)	26.4 ± 2.8 (21–30)	0.606
Operation time^*β*,*¥*^ (min)	58.6 ± 28.3 (20–110)	52.8 ± 18.7 (25–90)	0.476
Peritoneal injury (*n*/%)	7 (33.3)	2 (9.5)	0.130
Conversion (*n*/%)	7 (33.3)	0 (0)	0.009*

*Statistical significance.

^*β*^Mean ± SD.

^*¥*^Operation times excluding converted operations.
